# Effects of a piano music listening intervention on depression, academic burnout, and mindfulness: evidence from a randomized controlled trial among Chinese university students

**DOI:** 10.3389/fpsyg.2026.1745444

**Published:** 2026-07-09

**Authors:** Liheng Yang, Ruikai Yuan, Yu Zheng

**Affiliations:** 1 Faculty of Music, Yuncheng University, Yuncheng, China; 2 School of Foreign Languages, Southeast University, Nanjing, China; 3 Department of Electrical and Electronic Engineering, The Hong Kong Polytechnic University, Kowloon, Hong Kong SAR, China

**Keywords:** academic burnout, depression, mental health promotion, mindfulness, piano music listening, university students

## Abstract

**Introduction:**

University students are increasingly vulnerable to mental health problems, such as depression and academic burnout, due to academic pressure and social challenges. As a non-pharmacological and cost-effective approach, music-based interventions have shown promise in improving psychological well-being. This study aimed to evaluate the effectiveness of a short-term piano music listening intervention in improving depressive symptoms, mindfulness levels, and academic burnout among Chinese university students.

**Methods:**

A randomized controlled trial was conducted among 120 undergraduate students (drawn from 125 randomized; see participant flow), who were randomly assigned to either a piano music intervention group (*n* = 60) receiving daily piano music listening sessions for 20 days, or a white noise control group (*n* = 60). Data were analyzed using R software with mixed-design ANOVA.

**Results:**

Post-intervention analysis showed a significant reduction in depressive symptoms (*p* < 0.001) and academic burnout subscales (*p* < 0.05), as well as a significant increase in mindfulness levels (*p* < 0.001) in the intervention group compared to the control group.

**Conclusion:**

The findings suggest that a short-term piano music listening intervention may be beneficial for reducing depressive symptoms and improving mindfulness and academic burnout among university students under the conditions tested, and may serve as a complementary strategy for promoting student mental health in educational settings.

## 1 Introduction

The primary objective of a college education is to facilitate the intellectual growth of students and equip them with the necessary skills to lead successful and productive adult lives (Darling-Hammond et al., 2020). Nevertheless, students encounter novel obstacles during this transitional period, including the first time they are required to live independently, establish new friendships, and, for a significant number of them, depart from their homes and families (Naser et al., 2021). This can have a substantial effect on the physical, mental, and psychological well-being of students, resulting in issues such as anxiety, sleep disturbances, stress-related difficulties, and substantial psychological distress. Students’ academic achievements may be affected by these concerns (Shan et al., 2022).

To resolve the challenges encountered by college students, the university education system must promote the development of social–emotional competence and academic skills (Lee et al., 2023; Evans et al., 2018). Nonetheless, despite increasing focus, current research on the mental health of college students has neglected numerous critical domains. First, psychological counseling services are often prohibitively expensive, making them inaccessible to many students, particularly those from lower socioeconomic backgrounds (Singla et al., 2023). Second, traditional therapeutic interventions—such as cognitive behavioral therapy—tend to be time-consuming, resource-intensive, and often fail to accommodate the fast-paced, high-pressure lifestyles of undergraduate populations (Wei et al., 2024). As a result, there is an urgent need for alternative, scalable, and low-cost interventions that can effectively support students’ emotional well-being without requiring clinical supervision or formal diagnostic procedures.

Music is an art form that embodies authentic human emotions and serves as a medium for expressing individuals’ sentiments, communicating affect more directly than conventional language (Gaunt et al., 2021). Piano music listening, as a form of receptive music-based intervention, has been shown to support patients with physical or psychological conditions and has gained growing popularity as a supplementary approach for depression across diverse settings (Yang et al., 2021; Li et al., 2021). Nonetheless, a limited number of empirical investigations have concentrated on non-clinical university students. For example, Finnerty and Trainor (2025) conducted a group music therapy study focused on the proactive management of stress and anxiety in non-clinical undergraduate students, demonstrating the feasibility and benefits of music-based approaches in this population.

This study sought to examine the impact of a piano music listening intervention on depression, mindfulness, and academic burnout in Chinese university students using a randomized controlled trial. This study seeks to furnish empirical information about the utilization of receptive music-based approaches in psychological interventions for non-clinical groups and to enhance both theoretical and practical methodologies for mental health interventions among university students.

## 2 Literature review

### 2.1 Music-based interventions

Studies have examined music-based interventions as accessible, non-pharmacological approaches for improving mental health outcomes. Systematic reviews and meta-analyses have reported beneficial effects on depressive symptoms, stress, anxiety, and emotional well-being across clinical and non-clinical populations (Tang et al., 2020; Zhao et al., 2016; Rodwin et al., 2023). Notably, recent meta-analyses focusing specifically on college students have confirmed significant effects of music therapy on depressive symptoms (Lin and Li, 2025) and anxiety (Li et al., 2025) in this population. Numerous systematic evaluations have determined that music-based interventions can alleviate depressive symptoms in adults (Rodwin et al., 2023; Tang et al., 2020; Zhao et al., 2016; Lu et al., 2021; Maratos et al., 2011). Imani et al. (2021) demonstrated notable improvements in depression levels among hemodialysis patients, while Ye et al. (2021) revealed the function of music in relieving aggressive behavior in both children and adolescents. Erkkilä et al. (2021) implemented a randomized controlled trial finding that listening homework can augment therapeutic outcomes in individuals with depression. Hartmann et al. (2023) further suggested that musical interaction features may serve as markers for therapeutic progress.

### 2.2 Mindfulness

Mindfulness is an inherent human capacity that varies among individuals due to practice, life circumstances, and presumably genetic predispositions, which increase attention and awareness of the present moment (Baer et al., 2019). Although there is anecdotal evidence and a general consensus regarding the types of music that facilitate mindfulness practice (such as slow, repetitive, and legato compositions), empirical research examining the utilization and effects of music in mindfulness meditation remains in its infancy (Hernandez-Ruiz and Dvorak, 2021). Prior studies have found that complex melodic stimuli are favored and deemed more advantageous for mindfulness meditation compared to the absence of music or steady tones (Reybrouck and Brattico, 2022).

### 2.3 Academic burnout

Recent studies have identified multiple factors that may affect students’ psychological distress, including academic challenges, aspirations for success, adaptation to the university setting, and apprehensions regarding the future (Ramler et al., 2016; Finkelstein-Fox et al., 2018; Nardi et al., 2022). College students represent a susceptible demographic owing to the heightened likelihood of experiencing mental problems during early adulthood (Limone and Toto, 2022). The prevalence of depression among Chinese university students was found to be 28.4% (*n* = 185,787) (Gao et al., 2020). Psychological issues not only have significant repercussions for students, including heightened stress, diminished academic performance, and lowered quality of life, but also for university institutions.

Despite the growing attention to mindfulness and music-based interventions, several research gaps remain. First, most existing studies target clinical populations, leaving the mental health needs of non-clinical university students relatively underexplored. Chen et al. (2024) evaluated music-based interventions in non-clinical medical students, and Li et al. (2025) and Finnerty et al. (2023), who conducted systematic reviews and trials targeting stress and anxiety in non-clinical undergraduate samples. Second, while music and mindfulness share theoretical affinities, their empirical intersection remains limited. Third, the potential role of piano music listening interventions in simultaneously addressing depression, enhancing mindfulness, and reducing academic burnout has not been sufficiently examined through rigorous randomized controlled trials.

## 3 Method

### 3.1 Research design

This study employed a randomized controlled trial (RCT) design with a two-group, pretest–posttest structure to examine the effects of a piano music listening intervention on college students’ psychological well-being. Participants were randomly assigned to either an experimental group (piano music listening intervention) or a control group (white noise, WN). Both groups completed standardized self-report measures assessing mindfulness, depression, and academic burnout before and after the 20-day intervention period. The experimental manipulation (type of auditory stimulus) was the sole difference between groups. To enhance ecological validity, the intervention was designed for home-based implementation using audio recordings played via personal devices (e.g., headphones or speakers). Participants were instructed to listen during a designated relaxation window (20:00–22:00), though they were permitted to choose a specific time within that window. Each daily session lasted 20 min, using a standardized playlist of 12 solo piano pieces (see Appendix Table A1 for the full track list). Tracks were played sequentially without looping; playback stopped automatically at 20 min. Compliance was monitored through daily self-report logs; however, quantitative compliance data were not systematically aggregated across participants, which represents a limitation of intervention fidelity monitoring (see Section 5). White noise was selected as the control condition to control for the non-specific effects of auditory stimulation (e.g., time spent in quiet listening, masking of environmental noise) while avoiding the emotional or cognitive engagement elicited by musical structure.

### 3.2 Participants

**Figure 1:**
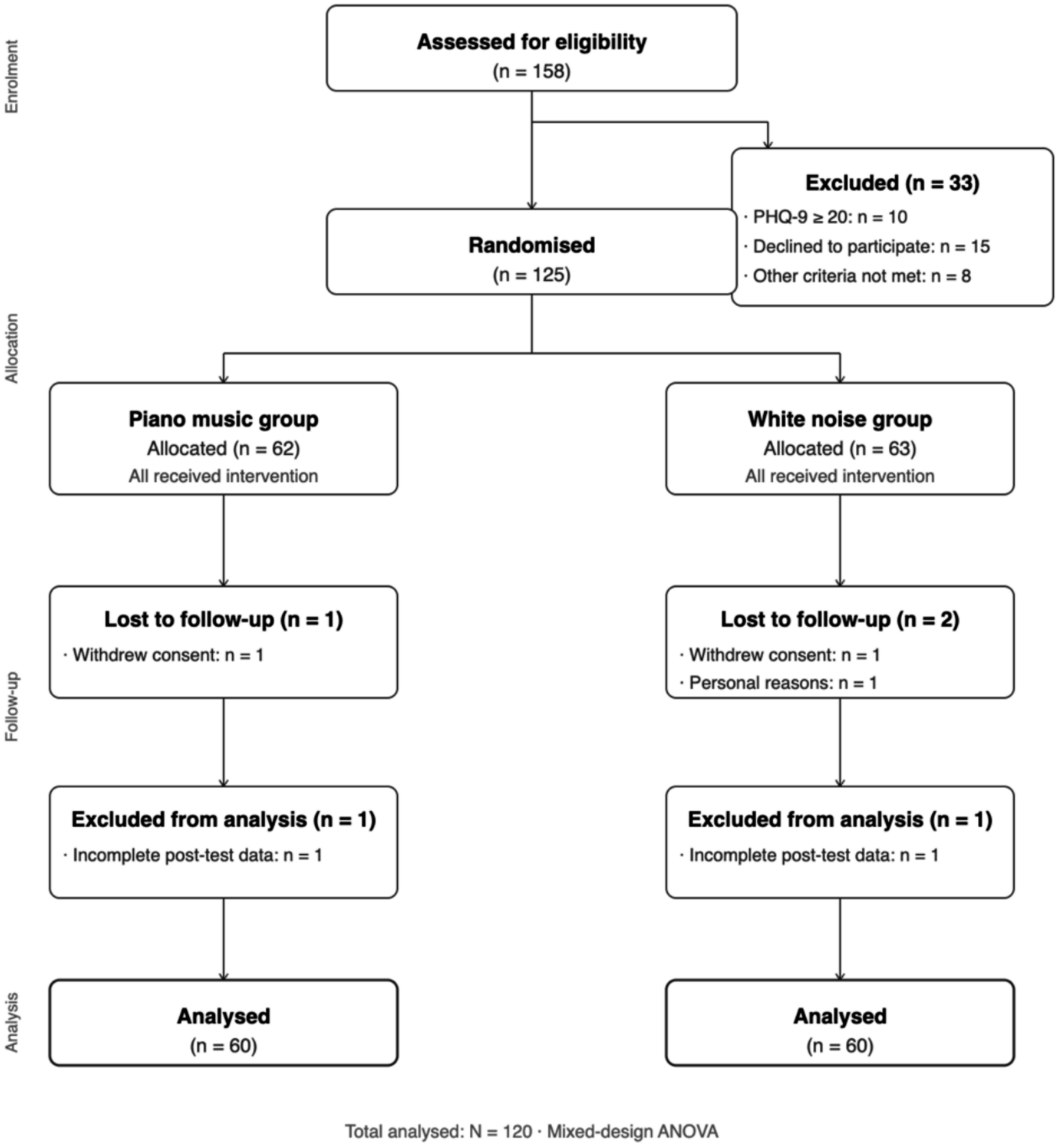
CONSORT flow diagram.

Sample size estimation was performed using G*Power 3.1, assuming a medium effect size (*f* = 0.25), *α* = 0.05, and power (1 − *β*) = 0.80 for a mixed-design ANOVA with two groups and two time points, indicating a minimum required sample of 102 participants. To account for potential attrition, we aimed to recruit approximately 125 participants, which would allow for up to 18% dropout while maintaining adequate statistical power. Participants were randomized to the piano music listening intervention group (*n* = 62) or the white noise control group (*n* = 63) using a computer-generated block randomization sequence (block size = 4). Group assignment was performed by a research assistant independent of the data collection and intervention processes, and allocation concealment was ensured using sealed opaque envelopes. The study adhered to the CONSORT guidelines for randomized trials in psychological and behavioral research (Figure 1).

Three participants were lost to follow-up during the intervention period (1 from the piano group, 2 from the white noise group). Two participants were excluded from analysis due to incomplete post-test/tracking data, one from each group. After excluding these participants, 120 participants with complete pre- and post-test data were included in the final analysis (piano group: *n* = 60; white noise group: *n* = 60). The final analysis was conducted as a complete-case analysis including participants with complete pre- and post-test data; no intention-to-treat analysis was performed. Given the low and similar attrition across groups and the absence of reported adverse events, attrition did not appear to be clearly differential between groups.

Participants were undergraduate students enrolled at Yuncheng University in Shanxi, China. Recruitment was conducted via WeChat groups. Eligible participants were between 18 and 25 years of age, enrolled as full-time undergraduate students regardless of academic major, and demonstrated mild to moderate depressive symptoms, defined as scoring between 5 and 19 on the PHQ-9. Participants were required to have no history of diagnosed psychiatric or neurological disorders, no current use of psychoactive medication, and no participation in music or mindfulness-based interventions within the past 6 months. Students with extensive formal music training (i.e., music majors) were excluded to reduce familiarity bias; those with basic musical exposure were included. Individuals with reported hearing impairment were also excluded. Those with PHQ-9 scores of 20 or above were advised to seek professional care and were not enrolled. All participants provided informed consent prior to their participation, and ethical approval for the study was granted by the Ethics Committee of Yuncheng University [ID: 20250315002].

The primary outcome of this study was depressive symptoms (PHQ-9). Secondary outcomes were mindfulness (MAAS) and the three subscales of academic burnout (MBI-SS: Emotional Exhaustion, Cynicism, and Academic Efficacy).

### 3.3 Measures

#### 3.3.1 Patient health questionnaire-9 (PHQ-9)

The PHQ-9 is a widely used, validated self-report instrument designed to screen for the presence and severity of depressive symptoms (Kroenke et al., 2001; Sun et al., 2022). It consists of nine items rated on a 4-point Likert scale ranging from 0 (“not at all”) to 3 (“nearly every day”), yielding a total score ranging from 0 to 27. In this study, only participants scoring between 5 and 19 were included. The PHQ-9 demonstrated acceptable internal consistency in the current sample (Cronbach’s *α* = 0.715).

#### 3.3.2 Maslach burnout inventory student survey (MBI-SS)

Academic burnout was measured using the validated Chinese version of the Maslach Burnout Inventory–Student Survey (MBI-SS; Hu and Schaufeli, 2009). The MBI-SS consists of 15 items across three subscales: Emotional Exhaustion (EE; 5 items), Cynicism (CY; 4 items), and Academic Efficacy (AE; 6 items). Items are rated on a 7-point Likert scale from 0 (“never”) to 6 (“every day”). Higher scores on EE and CY indicate higher burnout, whereas higher scores on AE indicate greater academic efficacy and therefore lower burnout. For interpretability, the AE subscale was reported in its original direction. If an overall burnout score is computed, AE items should be reverse-coded so that all items are aligned in the same direction. Cronbach’s alpha was computed separately for each subscale: EE *α* = 0.81, CY *α* = 0.78, AE *α* = 0.76. The overall 15-item internal consistency was *α* = 0.839.

*Note*: Throughout this manuscript, the Academic Efficacy subscale of the MBI-SS is abbreviated as AE, consistent with the original instrument. Earlier drafts used the abbreviation PE; this has been corrected throughout.

#### 3.3.3 Mindful attention awareness scale (MAAS)

The MAAS is a 15-item scale measuring dispositional mindfulness (Deng et al., 2012; Brown and Ryan, 2003). The validated Chinese version was used in this study. Items are scored on a 6-point scale; higher scores indicate greater mindfulness. In the current sample, internal consistency was acceptable (Cronbach’s *α* = 0.758).

### 3.4 Data analysis

All statistical analyses were conducted using R version 4.3.2 within the RStudio environment (Ihaka and Gentleman, 1996). Data visualization and descriptive statistics were performed using the ggplot2, dplyr, and emmeans packages. Prior to inferential testing, the data were screened for normality using Shapiro–Wilk tests and visual inspection of histograms and Q–Q plots. Some outcomes (PHQ-9 post-test and MBI-SS AE) showed mild departures from normality (*W* values ranging from 0.946 to 0.958, all *p*s < 0.05); however, given the sample size of *N* = 60 per group, mixed ANOVA is considered robust to mild violations of normality.

To assess the effects of the intervention, a 2 (Group: Piano vs. White Noise) × 2 (Time: Pre-test vs. Post-test) mixed-design ANOVA was conducted separately for each dependent variable. The analysis was performed using the afex package with Type III sums of squares and Greenhouse–Geisser correction where appropriate. For significant interaction effects, *post hoc* pairwise comparisons were conducted using the emmeans package with Bonferroni-adjusted *p*-values. Effect sizes were reported using partial eta squared (*η*^2^*p*) for ANOVA models and Cohen’s *d* for within-group pre–post comparisons. Ninety-five percent confidence intervals (CIs) were reported for *η*^2^*p* and for key pairwise mean differences to improve the precision and interpretability of findings. Given the significant baseline group difference on the MBI-SS Academic Efficacy (AE) subscale (*t* = 3.04, *p* = 0.003), an ANCOVA was additionally conducted for this outcome with baseline AE as the covariate.

## 4 Results

### 4.1 Descriptive data

The demographic characteristics of the participants are summarized in Table 1. The final sample consisted of 120 undergraduates, of whom 70 (58.3%) were female and 50 (41.7%) were male. Participants were distributed across all grade levels: 29 freshmen (24.2%), 34 sophomores (28.3%), 23 juniors (19.2%), and 34 seniors (28.3%). Chi-square tests confirmed that the two groups were comparable on all demographic variables (all *p*s > 0.18; see Table 1).

**Table 1:** Demographic characteristics of participants by group (*N* = 120).

Variable	Category	Piano *n*	Piano %	WN *n*	WN %
Gender	Female	34	56.7	36	60.0
	Male	26	43.3	24	40.0
Grade	Freshman	15	25.0	14	23.3
	Sophomore	19	31.7	15	25.0
	Junior	9	15.0	14	23.3
	Senior	17	28.3	17	28.3
Major	Arts	20	33.3	11	18.3
	Humanities	11	18.3	15	25.0
	Medicine	11	18.3	15	25.0
	Science	10	16.7	10	16.7
	Other	8	13.3	9	15.0
Residency	Yes	30	50.0	27	45.0
	No	30	50.0	33	55.0
Part-time job	Yes	23	38.3	28	46.7
	No	37	61.7	32	53.3
Psychological counseling	Never	14	23.3	23	38.3
	Occasionally	26	43.3	19	31.7
	Frequently	20	33.3	18	30.0

Chi-square tests revealed no significant group differences on any demographic variable (all *p*s > 0.18).

Pre- and post-test means and standard deviations by group are presented in Table 2. At pre-test, PHQ-9 scores were comparable between the Piano group (*M* = 10.73, SD = 3.86) and the White Noise group (*M* = 11.02, SD = 3.79). For MBI-SS subscales, the Piano group scored *M* = 2.88 (SD = 0.59) on EE, *M* = 2.95 (SD = 0.61) on CY, and *M* = 2.96 (SD = 0.59) on AE. The White Noise group scored *M* = 2.79 (SD = 0.68), *M* = 2.78 (SD = 0.67), and *M* = 2.63 (SD = 0.61), respectively. MAAS scores were virtually identical at baseline (Piano: *M* = 3.63, SD = 0.29; White Noise: *M* = 3.64, SD = 0.26). Baseline t-tests confirmed group equivalence for PHQ-9, MBI EE, MBI CY, and MAAS (all *p*s > 0.10); however, a significant pre-test difference was observed on MBI-SS Academic Efficacy (*t* = 3.04, *p* = 0.003, *d* = 0.55), with the Piano group scoring higher than the White Noise group.

**Table 2:** Pre- and post-test means (SD) and within-group effect sizes by group.

Outcome	Piano pre *M* (SD)	Piano post *M* (SD)	WN pre *M* (SD)	WN post *M* (SD)	Within-group *d* (piano)
PHQ-9	10.73 (3.86)	4.95 (3.53)	11.02 (3.79)	11.00 (3.87)	1.56
MBI-SS EE	2.88 (0.59)	2.30 (1.08)	2.79 (0.68)	2.86 (1.04)	0.67
MBI-SS CY	2.95 (0.61)	2.38 (1.32)	2.78 (0.67)	2.72 (1.39)	0.56
MBI-SS AE	2.96 (0.59)	3.44 (0.71)	2.63 (0.61)	2.71 (0.67)	0.75
MAAS	3.63 (0.29)	4.20 (0.61)	3.64 (0.26)	3.55 (0.43)	1.18

WN, white noise group. *d* = Cohen’s *d* for Piano group pre–post change.

### 4.2 Mixed ANOVA

As shown in Table 3, mixed-design ANOVAs were conducted for all five dependent variables. For depression (PHQ-9), a highly significant Group × Time interaction was found, *F* (1, 118) = 270.52, *p* < 0.001, *η*^2^*p* = 0.696, 95% CI [0.625, 1.000], indicating that the Piano group showed a substantially greater reduction in depressive symptoms than the White Noise group. For burnout, significant interactions were observed for Emotional Exhaustion, *F* (1, 118) = 19.28, *p* < 0.001, *η*^2^*p* = 0.140, 95% CI [0.057, 1.000]; Cynicism, *F* (1, 118) = 6.27, *p* = 0.014, *η*^2^*p* = 0.050, 95% CI [0.006, 1.000]; and Academic Efficacy, *F* (1, 118) = 29.76, *p* < 0.001, *η*^2^*p* = 0.201, 95% CI [0.104, 1.000]. For mindfulness (MAAS), the Group × Time interaction was significant, *F* (1, 118) = 66.04, *p* < 0.001, *η*^2^*p* = 0.359, 95% CI [0.249, 1.000]. Across all outcomes, the Piano group demonstrated more favorable changes from pre- to post-test. Note that the groups differed significantly on AE at baseline (*p* = 0.003), and this should be considered when interpreting post-test differences on that subscale.

**Table 3:** Mixed-design ANOVA results.

Outcome	Effect	d*f*	*F*	*p*	*η*^2^*p* [95% CI]
PHQ-9 (depression)	Group	1, 118	22.70	<0.001***	0.161 [0.057, 1.000]
	Time	1, 118	273.66	<0.001***	0.699 [0.638, 1.000]
	Group × time	1, 118	270.52	<0.001***	0.696 [0.625, 1.000]
MBI-SS emotional exhaustion (EE)	Group	1, 118	2.78	0.098	0.023 [0.000, 1.000]
	Time	1, 118	11.33	0.001**	0.088 [0.021, 1.000]
	Group × time	1, 118	19.28	<0.001***	0.140 [0.057, 1.000]
MBI-SS cynicism (CY)	Group	1, 118	0.26	0.612	0.002 [0.000, 1.000]
	Time	1, 118	9.67	0.002**	0.076 [0.015, 1.000]
	Group × time	1, 118	6.27	0.014*	0.050 [0.006, 1.000]
MBI-SS academic efficacy (AE)	Group	1, 118	22.75	<0.001***	0.162 [0.058, 1.000]
	Time	1, 118	60.65	<0.001***	0.339 [0.230, 1.000]
	Group × time	1, 118	29.76	<0.001***	0.201 [0.104, 1.000]
MAAS (mindfulness)	Group	1, 118	24.38	<0.001***	0.171 [0.064, 1.000]
	Time	1, 118	34.80	<0.001***	0.228 [0.120, 1.000]
	Group × time	1, 118	66.04	<0.001***	0.359 [0.249, 1.000]

*p < 0.05, **p < 0.01, ***p < 0.001. CIs for *η*^2^*p* were computed using the effectsize package; upper bounds of 1.000 reflect the asymmetric distribution of partial *η*^2^ and are expected for one-tailed confidence intervals.

### 4.3 Post-hoc comparisons

*Post hoc* pairwise comparisons with Bonferroni correction were conducted for each outcome (see Table 4). For PHQ-9, the Piano group showed a significant within-group reduction (Δ = 5.78, 95% CI [5.29, 6.27], *p* < 0.001, *d* = 1.56), while the White Noise group showed no meaningful change (Δ = 0.02, *p* > 0.99). The groups did not differ at pre-test (*p* > 0.99) but differed significantly at post-test (Δ = −6.05, 95% CI [−7.39, −4.71], *p* < 0.001), confirming baseline equivalence and a clear Group × Time difference for the primary outcome.

**Table 4:** Post-hoc pairwise comparisons (Bonferroni-corrected).

Outcome	Piano Δ [95% CI]	*p*	*d*	WN Δ [95% CI]	*p*	*d*	Between-group @Post Δ [95% CI]	*p*
PHQ-9	5.78 [5.29, 6.27]	<0.001***	1.56	0.02 [−0.48, 0.52]	>0.99	0.00	−6.05 [−7.39, −4.71]	<0.001***
MBI-SS EE	0.58 [0.37, 0.79]	<0.001***	0.67	−0.08 [−0.29, 0.14]	>0.99	—	−0.56 [−0.95, −0.18]	0.025*
MBI-SS CY	0.58 [0.37, 0.79]	0.001**	0.56	0.06 [−0.21, 0.34]	>0.99	—	−0.34 [−0.83, 0.15]	>0.99
MBI-SS AE	−0.49 [−0.59, −0.39]	<0.001***	0.75	−0.09 [−0.19, 0.02]	0.610	—	0.73 [0.49, 0.98]	<0.001***
MAAS	−0.57 [−0.68, −0.45]	<0.001***	1.18	0.09 [−0.03, 0.21]	0.707	—	0.65 [0.46, 0.84]	<0.001***

Δ, pre-test minus post-test mean difference. Between-group @Post Δ, piano post-test mean minus WN post-test mean. For PHQ-9, MBI EE, and MBI CY, positive Δ indicates reduction (improvement); for MAAS and MBI-SS AE, negative Δ indicates increase (improvement). *d* = Cohen’s *d* for Piano group pre–post change. *p < 0.05, **p < 0.01, ***p < 0.001.

For MAAS, the Piano group significantly improved (Δ = −0.57, 95% CI [−0.68, −0.45], *p* < 0.001, *d* = 1.18) while the White Noise group showed no significant change (Δ = 0.09, *p* = 0.707). For EE, the Piano group improved significantly (Δ = 0.58, 95% CI [0.37, 0.79], *p* < 0.001, *d* = 0.67) and the between-group post-test difference was significant (Δ = −0.56, 95% CI [−0.95, −0.18], *p* = 0.025). For CY, the Piano group improved (Δ = 0.58, 95% CI [0.29, 0.87], *p* = 0.001, *d* = 0.56), but the between-group post-test difference did not reach significance (*p* > 0.99).

On the AE subscale, the Piano group improved significantly (Δ = −0.49, 95% CI [−0.59, −0.39], *p* < 0.001, *d* = 0.75), and the between-group post-test difference was also significant (Δ = 0.73, 95% CI [0.49, 0.98], *p* < 0.001). However, given the pre-existing baseline imbalance (*t* = 3.04, *p* = 0.003), ANCOVA was conducted with baseline AE as the covariate. The adjusted group effect remained significant, *F* (1, 117) = 30.62, *p* < 0.001, adjusted Δ = 0.42, 95% CI [0.27, 0.58], with adjusted post-test means of 3.29 (Piano) vs. 2.87 (White Noise), confirming that the Piano group’s advantage on academic efficacy was not merely a function of pre-existing group differences.

**Figure 2:**
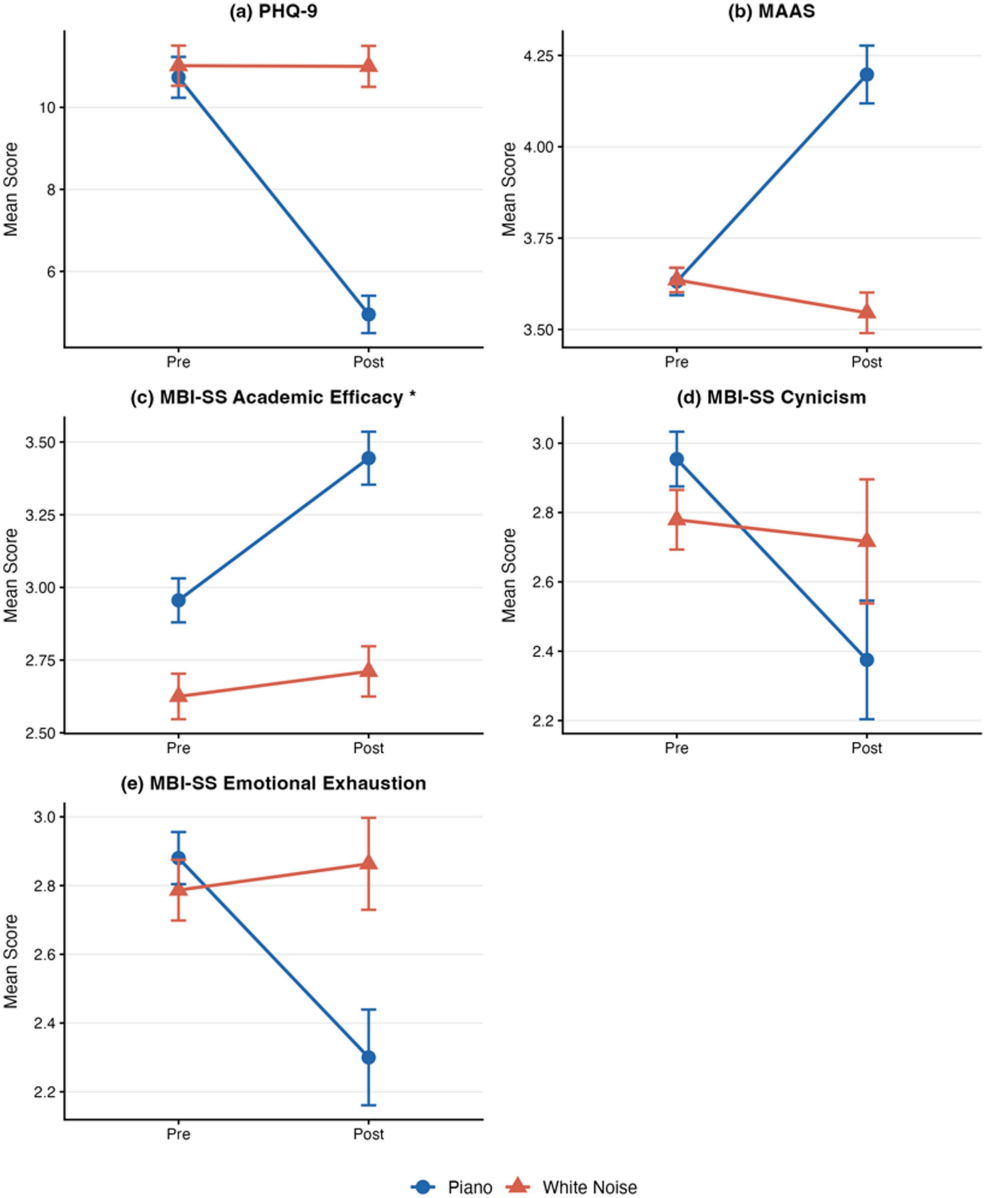
Mean scores on PHQ-9, MAAS, and MBI-SS subscales by group and time point. Error bars = ±1 SE. *Significant pre-test group difference on academic efficacy (c; *p* = 0.003); interpret with caution.

Figure 2 displays the mean scores across the five outcomes by group and time point.

## 5 Discussion

The present study investigated whether a short-term piano music listening intervention may reduce depression and academic burnout while improving mindfulness among Chinese undergraduate students. The findings demonstrated that participants in the piano music group exhibited significantly greater improvements in mindfulness levels and reductions in depression scores compared to the white noise control group. Specifically, the Group × Time interaction was significant across all five outcomes—depression, mindfulness, and all three dimensions of academic burnout. Effect sizes ranged from small-to-medium (*η*^2^*p* = 0.050 for Cynicism) to very large (*η*^2^*p* = 0.696 for PHQ-9), suggesting that piano music listening may offer meaningful psychological benefits for non-clinical university students.

Our findings align with prior research indicating the psychological benefits of music-based interventions. Several studies have emphasized the role of music in stress reduction and cognitive functioning in young adults (Nadon et al., 2021; De Witte et al., 2022). Research on mindfulness-based music listening has shown significant improvements in attention regulation (Liu et al., 2021) and emotional stability, which mirror the present study’s outcome on increased MAAS scores. Similarly, earlier trials by Wijeratne et al. (2022) have demonstrated that structured music listening can reduce depressive symptoms by promoting positive affect and a sense of calm.

The exceptionally large effect on depressive symptoms (*d* = 1.56, *η*^2^*p* = 0.696) is particularly noteworthy. PHQ-9 scores in the Piano group dropped from *M* = 10.73 to *M* = 4.95, representing a clinically meaningful transition from mild-to-moderate to subclinical levels of depression. In contrast, the White Noise group remained virtually unchanged across the intervention period (*M* = 11.02 to *M* = 11.00). The piano compositions used in the intervention were carefully selected to be lyrical, slow-paced, and free of lyrics—characteristics known to enhance affective engagement and facilitate mindfulness (Hernandez-Ruiz and Dvorak, 2021). From a neuropsychological perspective, such music may activate parasympathetic pathways and modulate limbic system activity, thereby lowering arousal and fostering introspective awareness (Barnett and Vasiu, 2024).

The strong effect on mindfulness (*η*^2^*p* = 0.359, *d* = 1.18) is consistent with theoretical accounts linking music and mindful attention. The MAAS score in the Piano group increased from *M* = 3.63 to *M* = 4.20, whereas the White Noise group showed negligible change (*M* = 3.64 to *M* = 3.55). This finding suggests that piano music may serve as an accessible anchor for present-moment awareness, particularly for individuals who find silent mindfulness practice challenging.

For academic burnout, all three subscales showed significant Group × Time interactions, although effect sizes differed substantially. EE (*η*^2^*p* = 0.140, *d* = 0.67) and AE (*η*^2^*p* = 0.201, *d* = 0.75) showed medium-to-large effects, while CY showed a smaller but still significant effect (*η*^2^*p* = 0.050, *d* = 0.56). Notably, the between-group post-test difference for CY was not significant (*p* > 0.99), suggesting that group-level differences in cynicism were no larger than what might be expected by chance after Bonferroni correction; this finding should be interpreted with caution. For AE, the ANCOVA with baseline covariate adjustment confirmed that the intervention effect was robust even after controlling for the pre-existing group imbalance, with adjusted post-test means of 3.29 vs. 2.87 in favor of the Piano group. The relatively smaller effect on CY compared to other outcomes may reflect the more entrenched and dispositional nature of attitudinal disengagement from academic work.

Nevertheless, not all prior studies have observed significant benefits from music interventions. Some investigations found null effects, particularly when using music with lyrics, complex rhythms, or in unstructured settings (Oliver et al., 2021). The inconsistency may stem from differences in musical genre, listening context, cultural familiarity, or participant engagement levels. In contrast, the current study’s controlled design likely contributed to the consistency of findings. Additionally, the use of white noise as the control condition was intended to isolate the effects of musical structure specifically. White noise controls for non-specific aspects of auditory stimulation—such as time spent listening in a quiet environment and the masking of ambient environmental noise—while precluding the emotional and cognitive engagement that musical structure elicits. This design choice strengthens the inference that observed group differences reflect the properties of piano music rather than auditory stimulation per se, though it also means that the absolute effects of white noise exposure (positive or negative) remain uncharacterized relative to a no-treatment baseline.

These findings suggest that piano music listening interventions may be beneficial for university mental health programs as a low-cost, scalable, and culturally acceptable supplement to traditional counseling services. University instructors and administrators may consider integrating brief music sessions into campus wellness activities as part of a broader burnout-prevention strategy.

## 6 Conclusion and limitations

The present findings suggest that a short-term piano music listening intervention may reduce depressive symptoms, improve mindfulness levels, and alleviate academic burnout among university students under the conditions tested. This type of structured, receptive music-based intervention may serve as a low-cost and accessible supplement to traditional mental health support in university settings. Given the rising prevalence of psychological distress among students and the limited accessibility of conventional counseling, integrating piano music listening into daily routines or campus wellness programs represents a promising avenue for supporting students’ emotional well-being. Attention to playlist standardization, listening environment, and session duration will be important for ensuring consistent effects in future applications.

Several limitations should be acknowledged. First, the short 20-day intervention duration may not capture long-term effects or sustainability of outcomes. Second, compliance was monitored via self-report daily logs; quantitative adherence rates were not systematically aggregated, which limits conclusions about intervention fidelity. Future research should employ objective compliance monitoring, registered protocols, longer follow-up periods, and more diverse samples to evaluate the durability and cross-cultural applicability of piano music listening interventions.

## Data Availability

The original contributions presented in the study are included in the article/supplementary material, further inquiries can be directed to the corresponding author.

## Appendix

**Table A1:** List of music used in the study.

No.	Name	Author	Time (cut)	Features
1	Clair de Lune	Claude Debussy	~5:00	Impressionistic harmony, flowing melody
2	Gymnopédie no. 1	Erik Satie	~3:30	Minimalist harmonic progression, meditative quality
3	Prelude in E minor, Op. 28 no. 4	Frédéric Chopin	~2:30	Lyrical melody, slow voice progression
4	Nocturne in Eb major, Op. 9 no. 2	Frédéric Chopin	~4:30	Typical nocturne form, rich in ornamentation
5	Intermezzo in A major, Op. 118 no. 2	Johannes Brahms	~5:00	Introspective quality, warm middle voice
6	Arabesque no. 1	Claude Debussy	~4:00	Flowing arpeggios, without strong rhythmic accents
7	Prelude in Db major, “Raindrop”	Frédéric Chopin	~5:00	Repeated bass lines, hypnotic effect
8	Traumerei (Kinderszenen Op. 15 no. 7)	Robert Schumann	~2:30	Nursery rhyme-like quality, simple binary form
9	Nocturne in C# minor, Posth.	Frédéric Chopin	~3:30	Tragic lyricism, profound
10	Gymnopédie no. 3	Erik Satie	~2:30	Slower than no. 1, almost still
11	Clair de Lune	Claude Debussy	~4:00	Deepens relaxation
12	Spiegel im Spiegel	Arvo Pärt	~6:00	Minimalism, mirrored structure, deep meditation
